# Tumor-associated macrophages in tumor metastasis: biological roles and clinical therapeutic applications

**DOI:** 10.1186/s13045-019-0760-3

**Published:** 2019-07-12

**Authors:** Yuxin Lin, Jianxin Xu, Huiyin Lan

**Affiliations:** 1 Department of Oncology, Hospital of Chinese Medicine of Changxing County, Huzhou, 313100 China; 2 Department of Radiation Oncology, Zhejiang Key Lab of Radiation Oncology, Zhejiang Cancer Hospital, Hangzhou, China; 3 Division of Radiation and Cancer Biology, Department of Radiation Oncology, University of Michigan, MS-1, 1301 Catherine Street, Ann Arbor, MI 48109 USA

**Keywords:** Metastasis, Macrophages, TAMs, TME, Polarization

## Abstract

Tumor metastasis is a major contributor to the death of cancer patients. It is driven not only by the intrinsic alterations in tumor cells, but also by the implicated cross-talk between cancer cells and their altered microenvironment components. Tumor-associated macrophages (TAMs) are the key cells that create an immunosuppressive tumor microenvironment (TME) by producing cytokines, chemokines, growth factors, and triggering the inhibitory immune checkpoint proteins release in T cells. In doing so, TAMs exhibit important functions in facilitating a metastatic cascade of cancer cells and, meanwhile, provide multiple targets of certain checkpoint blockade immunotherapies for opposing tumor progression. In this article, we summarize the regulating networks of TAM polarization and the mechanisms underlying TAM-facilitated metastasis. Based on the overview of current experimental evidence dissecting the critical roles of TAMs in tumor metastasis, we discuss and prospect the potential applications of TAM-focused therapeutic strategies in clinical cancer treatment at present and in the future.

## Introduction

Metastasis is a process of tumor cells escaping from the primary sites, spreading through lymphatic and/or blood circulations and ultimately disseminating to the distant sites. As one of the hallmarks of cancer, development of metastasis accounts for more than 90% cancer-related deaths [1]. Usually, the metastasis of tumor cells is a multistep sequence mainly including (a) invasion in the primary sites, (b) intravasation into the vasculature, (c) survival in the circulations, (d) extravasation out of the vasculature, and (e) adaption and growth in the metastatic sites [2, 3]. Failure in any of those steps will prevent the formation of metastasis. In addition to the alterations of the intrinsic properties in tumor cells, the “seed and soil” concept, firstly proposed by Stephen Paget in 1889, has been widely accepted as a critical theory to do with metastasis [4]. In this theory, tumor cells themselves are not sufficient for the development of metastasis. In fact, both the tumor cells and multiple components of the tumor microenvironment (TME) and their complicated cross talk are closely involved [5, 6]. Macrophages populating in the surrounding TME are usually termed as tumor-associated macrophages (TAMs) [7, 8]. A large volume of studies suggests that TAMs serve as prominent metastasis promoters in the TME, which orchestrate almost all of the 5 cascade steps of tumor metastasis as mentioned above [9, 10]. By producing growth factors, proteolytic enzymes, and various inhibitory immune checkpoint proteins in T cells, TAMs display implicated functions in regulating metastasis. Also, targeting TAMs as therapeutic strategies to prevent tumor progression and metastasis has attracted more and more researchers’ attention in recent years. So far, different types of molecular agents against TAMs are emerging as potential anti-cancer approaches. This review aims to provide an overview of the origin, classification, and polarization of TAMs as well as the mechanisms underlying the TAM-induced metastasis. Also, we will specifically discuss the agents targeting TAMs for cancer therapy. It is hoped that this review will help readers to understand the roles of TAMs in metastasis and their potential in clinic therapeutic applications against tumor progression.

## Overview: biological information and polarization of TAMs

### The definition, origin, and functions of TAMs

**Fig. 1:**
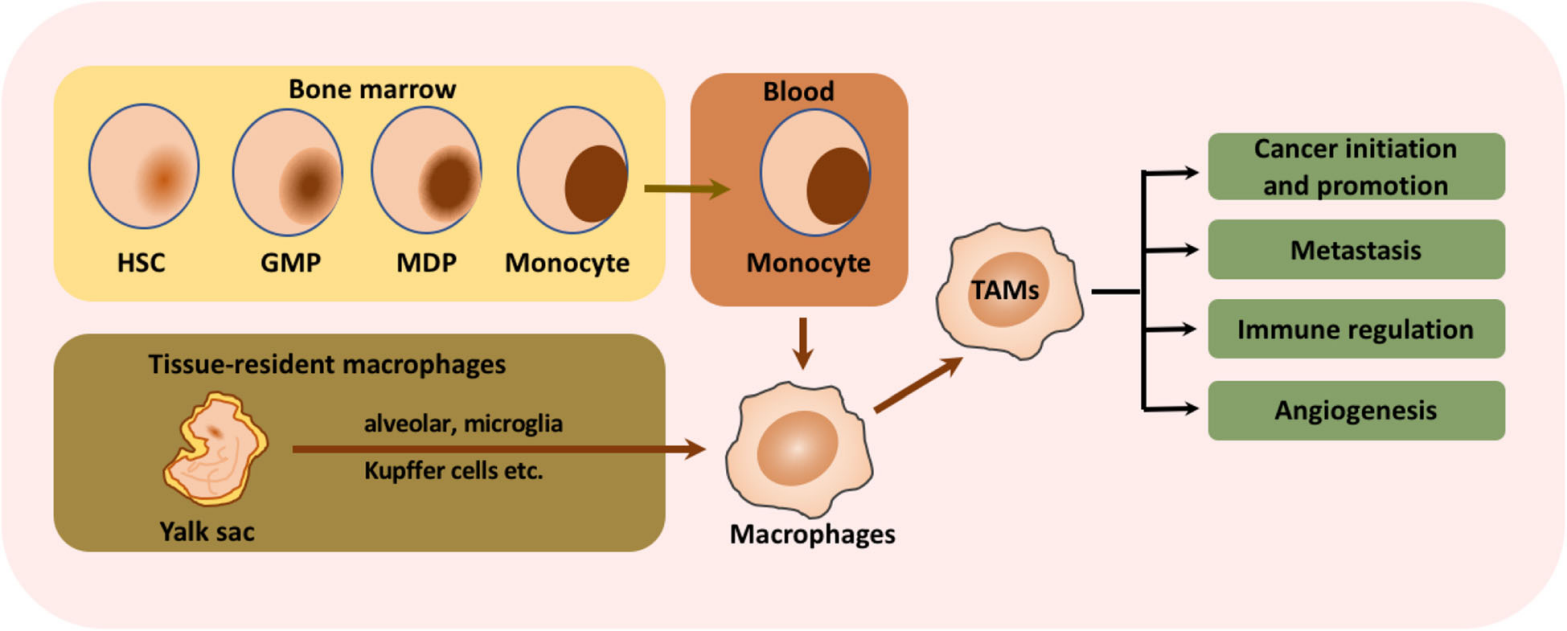
Cellular origins and functions of TAMs. As the major primary resource of macrophages, monocytes are generated from hematopoietic stem cells (HSCs) that differentiate into granulocyte-macrophage progenitors (GMPs) and then into monocyte-dendritic cell progenitors (MDPs). Besides, tissue-resident macrophage stem from yolk sac progenitors are another key resources of macrophages, which proliferate or differentiate in situ, such as alveolar macrophages, brain macrophages, and Kupffer cells. The mature monocytes released in the blood and tissue-resident macrophages are recruited and activated by various signals in the TME and then exhibit dramatic impacts on the tumor initiation and promotion, metastasis, immune regulation and angiogenesis

Macrophages are a type of versatile immunocytes, executing a broad spectrum of functions that range from modulating tissue homeostasis, defensing against pathogens, and facilitating wound healing [11]. Macrophages infiltrating tumor tissues or populated in the microenvironment of solid tumors are defined as tumor-associated macrophages (TAMs). As a critical component of tumor microenvironment, TAMs affect tumor growth, tumor angiogenesis, immune regulation, metastasis, and chemoresistance. Most of the TAMs gather in the leading edge and avascular areas, while some others align along the abluminal side of the vessels as well [12, 13]. It is generally believed that the blood monocytes derived from bone marrow hematopoietic stem cells are the primary resource of macrophages [14–16]. However, recent evidence suggests that a majority of resident macrophages stem from yolk sac progenitors, which proliferate or differentiate in situ and have progeny throughout their life, such as alveolar macrophages, brain macrophages, and Kupffer cells [11, 17–19]. They are recruited and activated by various signals in the TME and then exhibit dramatic impacts on the tumor progression and metastasis. The cellular origin of macrophages and TAMs was shown in Fig. 1.

Like macrophages perform diverse functions in immune regulation, TAMs also play multi-functional roles in tumor progression, including cancer initiation and promotion, immune regulation, metastasis, and angiogenesis, as shown in Fig. 1. For example, the presence of TAM-derived inflammatory cytokines interleukin (IL)-23 and IL-17 have been shown to trigger tumor-elicited inflammation, which in turn drives tumor growth [20] (Fig. 1). Another study demonstrated that the increased TAM-derived IL-6 exerts an amplifying effect on the inflammation response, thus promoting the occurrence and development of hepatocellular carcinoma via STAT3 signaling [21]. Moreover, TAMs acquire an M2-like phenotype, providing essential support on tumor progression and metastasis, despite their weak antigen presenting ability [22].

### The classification and polarization of TAMs

**Fig. 2:**
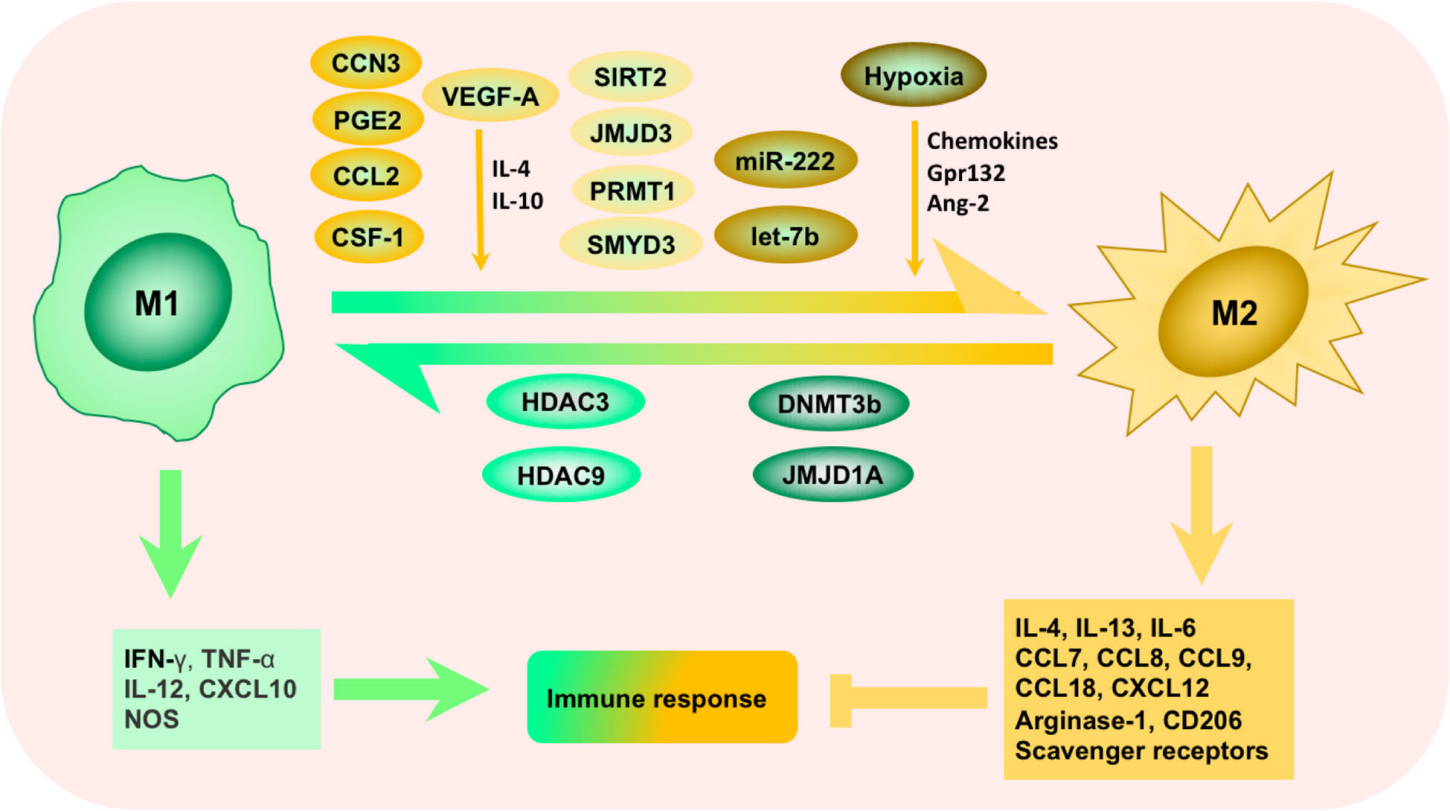
Tumor-associated macrophages (TAMs) polarization and its regulatory networks. Polarization of TAMs is regulated by multiple microenvironmental cytokines, growth factors, epigenetic regulators, and other signals derived from tumor and stromal cells. Two types of macrophages (M1/M2) secrete different immune markers, metabolic characteristics, and gene expression profiles to exert different functions

It is clear that macrophages are capable of displaying very different and even opposing phenotypes, depending on the microenvironment they embedded in. Activated macrophages are often classified into M1 (classical-activated macrophages) and M2 (alternative-activated macrophages) phenotype [23] (Fig. 2). In general, M1 macrophages foster inflammation response against invading pathogens and tumor cells, whereas M2 macrophages tend to exert an immune suppressive phenotype, favoring tissue repair and tumor progression. These two types of macrophages are distinct in their different markers, metabolic characteristics, and gene expression profiles. M1 macrophages secrete proinflammatory cytokines such as IL-12, tumor necrosis factor (TNF)-α, CXCL-10, and interferon (IFN)-γ and produce high levels of nitric oxide synthase (NOS, an enzyme metabolizing arginine to the “killer” molecule nitric oxide), while M2 macrophages secrete anti-inflammatory cytokines such as IL-10, IL-13, and IL-4 and express abundant arginase-1, mannose receptor (MR, CD206), and scavenger receptors [24, 25] (Fig. 2). The conversion between M1 (anti-tumorigenesis) and M2 (pro-tumorigenesis) is a biological process named “macrophage polarization” in response to microenvironmental signals [26]. Though studies found that TAMs are able to exhibit either polarization phenotype, researchers tend to consider TAMs as M2-like phenotype-acquired macrophages [22, 26–28]. It is consistent with these clinical observations that the accumulation of macrophages in the TME is largely associated with worse disease outcome [13, 29]. However, classification and identification of TAMs should be correlated mainly to their function such as metastasis, angiogenesis, and immune regulation. Expression of CD68, CD14, HLA-DR, and CD204 have been used for macrophage classification, and other proteins such as MMP2/9, B7-H4, STAT-3, CD163, and CD206 have been used for classification of TAMs [30]. We have listed these characterized biomarkers, CDs, and cytokines for TAM identification in Table 1. To better understand the correlation between TAMs, metastasis, and clinical applications in cancer therapy, we will further characterize the molecular mechanisms underlying TAMs polarization from M1-like to M2-like in detail below, also as shown in Fig. 2.

**Table 1:** Biomarkers associated with tumor-associated macrophages

Characteristics		Function	Expression		Detection		Ref.
			M1	M2	In situ	In vitro	
Biomarkers	MMP2/9	Matrix metalloproteinase	−	+	IHC	Digestion	[31]
	B7-H4	Inhibiting costimulatory molecule	−	+	IHC	Flowcytometry	[32]
	STAT-3	Transcription factor	−	+	IHC	Flowcytometry	[33]
	iNOS	Nitric oxide synthase	+	−	IHC	N/A	[34]
	HLA-DR	Antigen presentation molecule	+	+	IHC	Flowcytometry	[35]
CDs	CD68	Glycoprotein for adherence	+	+	IHC	Flowcytometry	[30]
	CD14	LPS co-receptor	+	+	IHC	Flowcytometry	[30]
	CD163	Scavenger receptor hemoglobulin	−	++	IHC	Flowcytometry	[30]
	CD206	Mannose receptor	+	++	N/A	Flowcytometry	[30]
	CD204	Macrophage scavenger receptor 1	+	+	IHC	N/A	[36]
Cytokines	IL-12p70	Interleukin	++	−	IHC	ELISA	[37]
	IL-10	Interleukin	+	++	IHC	ELISA	[37]

Marked with “−”: no expression; “+”: present on cell subset; “++”: highly expressed or produced

*IHC* immunohistochemical staining

Polarization of TAMs is regulated by multiple microenvironmental cytokines, chemokines, growth factors, and other signals derived from tumor and stromal cells [24]. Among those factors, colony stimulating factor 1 (CSF-1) and C-C motif ligand 2 (CCL2) are the most two well-documented macrophage recruiters and M2-stimulating factors (Fig. 2). CCL2 was earlier reported to shape macrophage polarization toward the protumor phenotype via the C-C chemokine receptor 2 (CCR2) expressed on the surface of macrophages [38]. Blocking the CCL2-CCR2 interaction either by genetic ablation or antibodies obviously inhibits metastatic seeding and prolongs the survival of tumor-bearing mice along with the diminished protumor cytokine expression [38–40]. Moreover, abundant clinicopathological data have verified the association between high concentrations of CCL2 in tumor with increased TAM infiltration and metastatic events [22, 39, 41]. CSF-1 is another potent determinant factor of macrophage polarization. CSF-1 wide overexpression is observed at the invasive edge of various tumors and correlates with a significant increase in metastasis [24]. In addition, tumor graft models showed that CSF-1 depletion led to greatly reduced macrophage density, delayed tumor progression, and severely inhibited metastasis [22, 24, 42, 43]. And the restoration of expression of CSF-1 in CSF-1 null mutant mice with xenografts accelerated both tumor progression and metastasis [42]. Vascular endothelial growth factor A (VEGF-A) has long been considered as a powerful pro-tumor factor [44]. Other than its pro-angiogenic effects, VEGF-A also fosters the malignant growth of tumors by inducing TAM infiltration and M2 polarization in the presence of IL-4 and IL-10 [45]. Direct evidence came from the gain-of-function experiments in the xenograft model of skin cancer, whereby VEGF-A upregulation rescued the clodronate induced macrophage depletion and resulted in shortened xenograft survival [45–47]. Besides, the overactivation of the epidermal growth factor receptor (EGFR) signaling pathway by either overexpression or mutation is frequently involved in tumor initiation, growth, and metastasis [48]. Actually, EGFR signaling not only promotes proliferation and invasiveness of tumor cells directly, but also adjusts the TME by regulating macrophage recruitment and M2-like polarization [49, 50]. Disrupted EGFR signaling by cetuximab or gene knockout resulted in less M2-polarized TAMs and correlated with better prognosis in colon cancer models of mice [51, 52]. Beyond those well-investigated factors mentioned above, a number of new homeostatic factors have been described as TAM inducers recently. For example, prostaglandin E2 (PGE2) synergized with CSF-1 to promote M2 polarization by transactivating the CSF-1R, and PGE2-elicited macrophage infiltration was significantly halted in the absence of CSF-1R [53]. In addition, CCN3 (also known as NOV, nephroblastoma overexpressed) led to enhanced M2 macrophage infiltration, whereas CCN3 deficiency prolonged xenograft survival in prostate cancer [54]. Furthermore, other chemokines such as IL-4, IL-6, IL-13, CCL7, CCL8, CCL9, CCL18, and CXCL12 are also highly expressed in tumors and involved in TAM recruitment and polarization [9, 10, 55–57] (Fig. 2).

Hypoxia, which resulted from tumor cells with a status of vigorous metabolism and rapid growth but poorly organized vasculature, is a common feature occurring in the majority of solid tumors [58]. Hypoxia promotes the malignant tumor behaviors by various mechanisms, such as inducing immune escape, promoting glycolysis, antagonizing apoptosis, promoting cell dedifferentiation, and reducing therapeutic effectiveness [59–61]. It is worth noting here that hypoxia also roles as a vital regulator of macrophages, which helps tumor cells overcome nutritive deprivation and convert the TME into more hospitable sites [28]. The gradients of chemokines induced by hypoxia, such as CCL2, CCL5, CSF-1, VEGF, semaphorin 3A (SEMA3A), endothelial cell monocyte-activating polypeptide-II (EMAP-II), endothelin, stromal cell-derived factor 1α (SDF1α), eotaxin, and oncostatin M, are responsible for the migration of TAMs into the hypoxic areas [28]. Hypoxia further traps the seeding macrophages by downregulating the chemokine receptors expressed on macrophages [62, 63]. Besides, hypoxia modulates the TAM phenotype toward a pro-tumoral profile by various factors. Lactate, massively produced by anaerobic glycolysis of tumor cells in oxygen-deprived areas, is one of the key inducers of M2 phenotype. It can be sensed by G protein-coupled receptor 132 (Gpr132), a membrane receptor on macrophages, which subsequently activates downstream signals and modulates the expression of polarization-associated genes [64]. And it has been shown that the enhanced expression of Gpr132 relates to the worse outcome of breast cancer patients, which was further verified by the positive association between the Gpr132 level and M2 macrophages infiltration, metastasis, and poor prognosis in breast cancer models in mice [64]. Similar stimulatory functions on macrophage accumulation and polarization can also be achieved by angiopoietin-2 (Ang-2), which is generally accepted as a regulator of vessel stabilization and growth in accompany with VEGF, Ang-1, via specifically binding to the receptor Tie-2 [65, 66] (Fig. 2). Ang-2 can also be dramatically upregulated by hypoxia [65]. However, there exists opposed evidence claiming that hypoxia is not the major driver of M1-M2 skewing [28, 67]. Instead of a direct effect on M2 transforming, hypoxia only fine-tunes hypoxia-regulated genes expression without influencing their M2 markers expression or the relative abundance of TAM subsets [67].

Epigenetic derangements is another universal feature in cancer. Epigenetic regulators reshape chromatin structures, pack the genome, and change gene expression patterns without altering the genome itself [68, 69]. More recently, a growing number of publications focus on the epigenetic participation in macrophage phenotypic switch [70, 71] (Fig. 2). Usually, most of the key points of epigenetic regulators are enzymes, which are druggable and easy to be translated into clinical applications for tumor intervention. For example, protein arginine methyltransferase 1 (PRMT1), SET and MYND domain-containing protein 3 (SMYD3), Jumonji domain-containing protein 3 (JMJD3), NAD-dependent protein deacetylase sirtuin-2 (SIRT), and bromodomain and extraterminal (BET) proteins positively regulate M2 polarization by upregulating M2 markers, while DNA methyltransferase 3b (DNMT3b), Jumonji domain-containing protein 1A (JMJD1A), histone deacetylase 3 (HDAC3), and HDAC 9 do the opposite effect [70, 71]. Interfering these epigenetic enzymes with pharmacologic modulators was able to prevent these macrophages from polarizing to M2 s and control the malignant progression of tumors.

As another type of epigenetic regulator, microRNAs (miRNAs) are also in control of macrophage polarization (Fig. 2). To date, miR-125, miR-155, miR-378, miR-9, miR-21, miR-146, miR-147, miR-187, miR-222, and miR-let7b have been reported as dominant TAM modulators [72]. For example, miR-222-3p, implicated as a tumor promoter in diverse tumor types, activates macrophages to the M2 phenotype by downregulating suppressor of cytokine signaling-3 (SOCS3) which is a negative feedback regulator of the JAK/STAT signaling pathway [73]. What is more, let-7b, enriched in prostatic TAMs, is drawing attention along the same line. Prostatic TAMs treated with let-7b inhibitors displayed characteristics of M1, with a significantly higher expression of pro-inflammatory cytokines (such as IL-10, IL-12, and IL-23), and downregulated pro-tumoral cytokines such as TNF-α [74].

Taken together, the polarization of TAMs is regulated by complicated biological networks (Fig. 2), which clinically correlates with cancer metastasis and progression.

## Mechanisms underlying TAM-facilitated metastasis

**Fig. 3:**
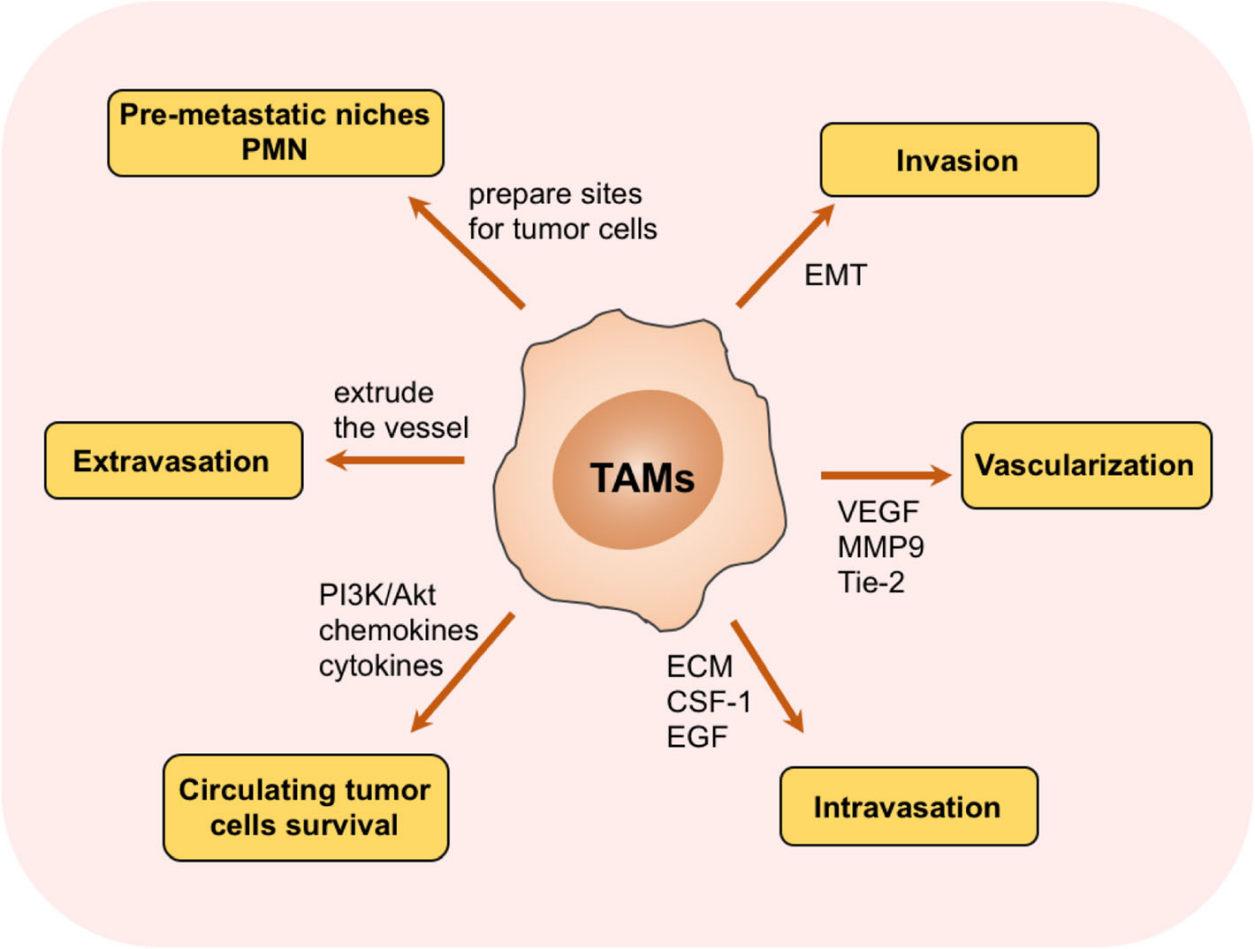
Mechanisms of tumor-associated macrophages (TAMs) in tumor metastasis. TAMs affect virtually almost every step of tumor cells metastasis, including invasion, vascularization, intravasation, extravasation, establishing pre-metastatic niches, and protecting circulating tumor cells survival

As mentioned above, TAMs display lots of important biological functions in tumor progression from different aspects. Here, we mainly focus on the correlation between TAMs and tumor metastasis. In fact, how TAMs contribute to tumor metastasis is a puzzling question which enables researchers to pursue the answers for dozens of years, though the existing studies demonstrate that TAMs implicate in almost every step of metastasis as described below, also shown in Fig. 3.

### TAMs promote invasion of tumor cells

Metastasis begins with tumor cells obtaining the ability of invasiveness and escaping from the confines of the basement membrane into the surrounding stroma [5, 75]. Highly invasive tumor cells always share the characteristics of loss of intrinsic polarity and loosely attachment to the surrounding tissue structures [76]. Epithelial-mesenchymal transition (EMT) is a predominant event in this morphological transformation, which contributes to malignant biological properties including invasion and metastasis [76]. During EMT process, tumor cells lose cell-cell junctions and apical-basal polarity as a result of E-cadherin repression and acquire a motile mesenchymal cell phenotype [77, 78].

Recently, a number of studies suggested that TAMs involve in the regulation of EMT process [79–81]. Immunostaining of clinical hepatocellular carcinoma (HC) samples revealed that the EMT hotpots, such as the edge of tumor nests, are also the sites where TAMs infiltrate in abundance [80]. Moreover, co-cultured HC cell lines with TAMs enhanced the expression of N-cadherin and Snail, both of which are hallmarks of mesenchymal phenotypes. Meanwhile, E-cadherin was observed to be downregulated. This phenomena also occurred in gastric cancer and pancreatic ductal adenocarcinoma (PDAC) [82]. Biologically, macrophages participate in the EMT process via secreting various soluble factors, such as IL-1β, IL-8, TNF-α, and transforming growth factor-β (TGF-β) [80, 83, 84]. Extracellular matrix (ECM) serves as a scaffold as well as a barrier for tumor cell migration [85], of which degradation is a focal event in metastasis. It has been identified that TAMs are capable of secreting a number of proteolytic enzymes, including cathepsins, matrix metalloproteinases (MMPs, such as MMP7, MMP2, and MMP9), and serine proteases, which are important components mediating ECM degradation and cell-ECM interactions [86–88]. In addition, an earlier study demonstrated that M2 macrophage promotes the invasiveness of gastric and breast cancer cells by producing chitinase 3-like protein 1 (CHI3L1). CHI3L1 upregulates MMP expression via interacting with interleukin-13 receptor α2 (IL-13Rα2) chain which triggers the activation of the mitogen-activated protein kinase (MAPK) signaling pathway [89]. Once the tumor cells break away from the constraint of ECM networks, they would move toward the stimuli along with the ECM fiber by interacting with other ECM components, such as fibronectin and vitronectin [90, 91]. Furthermore, secreted protein acidic and rich in cysteine (SPARC) synthesized by TAMs were shown to be necessary for the migration of tumor cells, aside from its role as an ECM deposition regulator. According to the earlier studies, SPARC favors fibronectin and vitronectin interaction with tumor cells through integrins, generating a traction force along ECM fibers [92, 93]. The traction force pulls tumor cells to rapidly travel through the stroma like tram lines and guarantees the rapid motivation of cells within stroma as well as toward tumor vasculature since many of those ECM fibers terminally converge on blood vessels [90]. Genetic ablation of SPARC led to attenuated metastasis by decreased ECM deposition and impaired tumor cell-ECM interaction [90, 92, 93].

### TAMs promote vascularization of tumor cells

Tumor vasculature serves as a major route for the metastasis of malignant tumors. When solid tumors grow up to a certain size, a process termed as “ angiogenic switch” will be turned on by various mechanisms to trigger a high-density vasculature for nutrients supply and wastes removal [94, 95]. TAMs are critical players in the regulation of “angiogenic switch.” They form clusters in the intra-tumoral regions and the invasive fronts, both of which are the hotspots of angiogenesis and metastasis. In contrast, the absence of TAMs significantly reduced the vessel density by 40% [96, 97]. In addition to affecting the formation of new tumor vessels, TAMs also stimulate the remodeling of the established vasculature to a more tortuous and leaky form in favor of tumor dissemination [96, 97]. In fact, researches strongly argue the important roles for VEGF and MMP-9 (plays a character in releasing VEGF from matrix) in regulating TAM-driven angiogenesis. Also, there are some other proangiogenic molecules involved as well, such as fibroblast growth factor (FGF)-2, CXCL8, IL-1, IL-8, cyclooxygenase (COX)-2, nitric oxides (iNOS), and MMP7 [96–99]. Furthermore, there is a novel subset of TAMs expressing tyrosine-protein kinase receptor Tie-2 (also known as angiopoietin-1 receptor) termed as TEMs [65, 100]. Experiments in a variety of tumor models clarify that TEMs were endowed with dramatic proangiogenic activity, since Tie-2 is capable of binding with all the known angiopoietins (Angs, including Ang-1, Ang-2, Ang-3, and Ang-4) [12, 65, 66]. Therefore, selective elimination of TEMs by a suicide gene strategy may be another promising option for preventing angiogenesis and tumor progression [66].

Besides, TAMs also account for lymphangiogenesis, an important route for tumor cells disseminating to regional lymph nodes and distant metastasis, in a VEGF-C (a ligand overexpressed by tumors)/VEGFR-3 (a receptor of VEGF-C expressed on the TAMs) axis-dependent manner. VEGF-C/VEGFR-3 axis fosters lymph angiogenesis either by directly affecting the lymphatic endothelial cells (LECs) activity or indirectly elevating the cathepsins secretion whose downstream molecular heparanase is a robust inducer of lymphangiogenesis [101–103]. From the mouse models, treatment with antibodies against VEGF-C/VEGFR-3 or genetic ablation of heparanase significantly altered the lymphatic vessel phenotype and subsequently impaired the primary tumor growth and metastasis [101].

Taken together, these evidences demonstrate that TAMs function in the way of promoting the vascularization of tumors via different pathways and thus are closely involved in tumor metastasis.

### TAMs promote intravasation of tumor cells

Tumor cells squeezing through small pores in vascular endothelium to gain access to the host vasculature is another critical step in metastasis [104]. An experiment utilizing intravital multiphoton imaging gave a direct and kinetical visualization of intravasation. According to this experiment, an intravasating tumor cell is always visualized to be accompanied by a macrophage within one cell diameter, showing a direct evidence of TAMs involving in tumor cell intravasation [105, 106]. Consistently, clinical observations have identified the tripartite arrangement of TAMs, tumor cells, and endothelial cells as the tumor microenvironment of metastasis (TMEM). The TMEM is a predictor of increased hematogenous metastasis and poor prognosis, at least in breast cancer [107]. The mechanisms underlying this synergistic interaction are complicated. On the one hand, macrophages break down the ECM around the endothelium by a number of proteolytic enzymes such as cathepsins, matrix metalloproteinases, and serine proteases [86–88]. On the other hand, TAMs hijack tumor cells into the circulation by a positive feedback loop consisting of tumor cell-produced CSF-1 and TAM-produced EGF [108]. The former cytokine stimulates macrophage’s motility as well as EGF production, which in turn signals to tumor cells and mediates chemotactic migration toward blood vessels [108, 109]. Therefore, inhibition of either CSF-1 or EGF signaling pathway perturbs the migration of both cell types and reduces the numbers of circulating tumor cells as well.

### TAMs promote tumor cell survival in the circulation

Once penetrated into the vasculature, the tumor cells have to be primed for survival and egress from the circulation. Clots packed around the tumor cells alleviate survival stress from such as natural killer (NK) cells in a tissue factor (TF)-dependent manner in the general circulation and capillaries [110, 111]. In fact, a strategy disrupting macrophage functions by genetic methods diminished the tumor cells survival in pulmonary capillaries and abrogated tumor invasion into the lung, despite clot formation, indicating an essential role of macrophages in this aspect [112]. Two plausible mechanisms might account for this phenomenon. In part, a recent study discovered that the recruited macrophages triggered the PI3K/Akt survival signaling pathway in newly disseminated breast cancer cells by engaging vascular cell adhesion molecule-1 (VCAM-1) via α4 integrins [113, 114]. The activation of the PI3K/Akt survival pathway subsequently saved cancer cells from proapoptotic cytokines such as TNF-related apoptosis-inducing ligand (TRAIL) [113]. In another part, many of the tumor cells survive which are protected by macrophages due to their secreted chemokines or cytokines directly secreted [112].

### TAMs promote extravasation of tumor cells

Once the tumor cells settle in the capillaries of the targeted organs, they would try to attach and extrude through the vessel walls with the assistant of macrophages. The intimate contacts between tumor cells and macrophages during extravasation were visualized and quantitatively analyzed within an intact lung imaging system [115]. Of particular importance, the researchers found that the extravasation rate was dramatically declined after the loss of macrophages together with a co-incident failure of metastasis [115].

### TAMs prepare sites for tumor cells: pre-metastatic niches (PMN)

It is believed that metastasis is not necessary to be a late event in tumor progression [116]. The primary tumors are smart enough to “prime” the secondary organs and dictate organ-specific dissemination before the arrival of tumor cells. Those “primed” sites are predisposed to metastasis and introduced as the concept of pre-metastatic niches (PMNs) [116]. Studies clarified that macrophages were one of the key determinants for the formation of PMNs. They were mobilized to the bloodstream and then clustered in the pre-metastatic sites by a variety of tumor-secreted factors, such as CCL2, CSF-1, VEGF, PLGF, TNF-α, TGF-β, tissue inhibitor of metallopeptidase (TIMP)-1, and exosomes [116–118]. Besides, the tissue-resident macrophages, such as liver Kupffer cells, pulmonary alveolar macrophages, and osteoclasts, were also involved in orchestrating PMN formation upon stimulation [119, 120]. The presence of those macrophages provide a road map for the homing of circulating tumor cells (CTCs) into the PMNs with enhanced expression of chemokines such as stromal derived factor (SDF)-1 and Ang-1 and remodel the ECM to the tumor cell-favoring direction by secreting ECM-shaping enzymes like MMPs, integrins, and lysyl oxidase (LOX), most of which have been mentioned above as critical inducers of angiogenesis, EMT, and extravasation [118–121]. Furthermore, macrophages also establish metabolic cross talk with immune cells like T helper 1 (TH1) cells and dendritic cells and attenuate their tumoricidal and tumor antigen-presenting behaviors, ultimately promoting the prosperity of those newly lodged tumor cells in a way of immunosuppression.

## Potential strategies targeting macrophages

Cancer is one of the most life-threatening diseases as a major public health problem with extremely high incidence and mortality all over the world. The progression in anti-tumor research never stops. While most of the therapeutic approaches nowadays mainly focus on malignant cells themselves, only limited efficiency has been achieved. However, in-depth knowledge of the cross talk between tumor cells and TME has reoriented our approaches to strategies against pro-metastatic non-tumor components in the TME. As described above, TAMs are one of the most essential accessory cells promoting the tumor progression and metastasis by various mechanisms. More importantly, TAMs are subject to the regulation of complicated molecular signals/factors, including lots of druggable enzymes and immune checkpoint proteins. As such, therapeutic approaches targeting TAMs are anticipated to be feasible and promising. Overall, the TAM-targeted therapeutic solutions would mainly focus on strategies to eliminate TAMs, impairing macrophages infiltration and suppressing phenotype conversion of M2 from M1 [82]. Next, we will discuss the current agents based on different mechanisms including inhibiting TAMs survival, suppressing M2 polarization and inhibiting macrophages recruitment as below, and we list these related agents in Table 2.

**Table 2:** Clinical trials of agents targeting TAMs for cancer treatment

Compound	Target	Combination partner	Tumor type	Phase	Status/results	Ref. or trial no.
Agents that inhibit TAM survival						
Trabectedin	Pan-macrophages	Durvalumab	Solid tumors	1	Not yet recruiting	NCT03496519
		Monotherapy	Mesothelioma	2	Recruiting	NCT02194231
Lurbinectedin (PM01183)	Pan-macrophages	Monotherapy	Solid tumors	1	No clinical consequences	[122]
		Monotherapy	Ovarian cancer	1	Active, not recruiting	[123]
		Gemcitabine	Solid tumors	1	CR, 3% PR, 21% PFS, 4.2 m	[124]
Agents that polarize TAMs to M1 type						
Zoledronic acid (ZA)	N/A	Monotherapy	Breast cancer	3	Prolonged survival	[125]
		Monotherapy	Breast cancer	2	Recruiting	NCT02347163
CP-870, 893	CD40	Monotherapy	Solid tumors	1	PR, 14%	[126]
		Gemcitabine	Pancreatic cancer	1	ORR, 19% PFS, 5.6% OS, 7.4%	[127]
Agents that inhibit TAM recruitment						
Emactuzumab (RG7155)	CSF-1R	Monotherapy	Solid tumors	1	PMR, 11% ORR, 0% CBR, 24%	[128]
		Monotherapy	Dt-GCT	1	CR + PR, 86% SD, 11%	[129]
		Atezolizumab	Solid tumors	1	Recruiting	NCT02323191
		Paclitaxel	Ovarian cancer Breast cancer	1	Not yet reported	NCT01494688
		Paclitaxel	Ovarian cancer	2	Active, not recruiting	NCT02923739
Pexidartinib (PLX3397)	CSF-1R	Monotherapy	Dt-GCT	2	PR, 52% SD, 30% PD, 4%	[130]
		Paclitaxel	Solid tumors	1	Not yet reported	NCT01525602
		Durvalumab	Colorectal cancer Pancreatic cancer	1	Recruiting	NCT02777710
		Monotherapy	Melanoma	1/2	Active, not recruiting	NCT02975700
		Monotherapy	Dt-GCT GCT-TS	3	Active, not recruiting	NCT02371369
ARRY-382	CSF-1R	Monotherapy	Solid tumors	1	ORR, 0% SD, 15%	[131]
		Pembrolizumab	Solid tumors	1b/2	Recruiting	NCT02880371
CCX872	CCR2	FOLFIRINOX	Pancreatic cancer	1b	18 m OS, 29%	[132, 133]
PF-04136309	CCR2	FOLFIRINOX	Pancreatic cancer	1b	ORR, 49%	[134]
Carlumab	CCL2	Monotherapy	Solid tumors	1b	Antitumor activity	[135]
		Monotherapy	Prostate cancer	2	No antitumor activity	[135]

### Agents against TAMs survival

Trabectedin is an agent with such cytotoxic efficacy to TAMs in TME; it has been approved for the treatment of patients with soft tissue sarcoma in Europe [136]. And it is also under clinical evaluation for other cancer types, including breast, prostate, and ovarian cancer [136]. Specifically, trabectedin is accepted as the cytotoxic agent directly killing tumor cells by interfering with several transcription factors, DNA-binding proteins, and DNA repair pathways [137]. Besides, its effects on the tumor microenvironment by selective mononuclear phagocyte depletion has been claimed as another key component of its antitumor activity [136]. Mechanically, trabectedin selectively induces rapid apoptosis in macrophages via TRAIL receptors and blocks their production of some pro-metastatic cytokines like CCL2, CXCL8, IL-6, and VEGF [136, 138]. The pro-apoptotic efficiency of trabectedin has been evaluated in a prospective study in which 56% (19 in 34) of soft tissue sarcoma patients experienced monocyte reduction with the extent ranging from 30~77% [136, 138]. Likewise, lurbinectedin (PM01183) is another novel anticancer agent structurally related to trabectedin. It functions by both directly killing tumor cells and affecting TAM-based immunomodulation [139]. As an analog of trabectedin, lurbinectedin exhibits potent apoptotic capacity upon macrophages, and by doing so, it dramatically decreases the number of macrophages both in circulation and TME in mice models [139]. Moreover, in the cancer cells resistant to chemotherapeutic agents, angiogenesis and distant dissemination were impaired due to lurbinectedin-caused macrophage depletion [139]. For clinical trials, various types of solid tumors in different programs are being conducted to evaluate the clinical benefits of lurbinectedin [122–124, 140–142]. However, both trabectedin and lurbinectedin cannot avoid the side effects arisen by unselectively macrophage consumption since macrophages closely participated in host defense and homeostatic regulation [140]. Thus, developing agents preferentially targeting M2-like macrophages is the “Holy Grail” to minimize potential toxic side effects. M2 macrophage-targeting peptide (M2pep), just as implied by the name, is such a construct discovered recently [143]. Researchers found that M2pep was able to exert selective toxicity to both tumor cells and M2 macrophages without influence on M1 macrophages both in vitro and in mice models [144, 145]. Based on these studies, M2pep has been turned out to be a promising adjuvant strategy for anticancer therapies, though it is still in the initial stage and needs a long way to go for substantial clinical applications.

### Agents suppressing M2 polarization and enhancing M1 activity of macrophages

As described above, it is widely believed that M2 and M1 macrophages play opposite roles in tumor growth and metastasis. Therefore, proposing therapeutic strategies re-educating the pro-tumor M2 phenotype into tumoricidal M1 phenotype and thus inhibiting TAMs’ supportive roles in tumors is feasible [146]. Zoledronic acid (ZA) is an eligible agent of this kind, which has been FDA-approved as the third generation of amino-bisphosphonate agent for treating skeletal-related events (SREs) and pain caused by bone metastasis. Beyond the skeleton, plenty of studies have generated new insights into its potent role in modulating macrophages phenotypes [147]. According to those studies, ZA was able to reverse the polarity of TAMs from M2-like to M1-like by attenuating IL-10, VEGF, and MMP-9 production and recovering iNOS expression [99, 148]. Furthermore, ZA was also capable of reducing the total number of macrophages in the TME by halting TAM recruitment and infiltration [149]. Based on this evidence, zoledronic acid has been added into the adjuvant endocrine therapy for premenopausal women with early-stage breast cancer in ABCSG-12 trial [125]. Data of 62 months’ follow-up [125] showed that the addition of ZA at clinically achievable doses delayed tumor recurrence and significantly prolonged disease-free survival, which provides a solid clinical evidence for ZA to be a promising agent for cancer prevention [147, 148]. Another agent capable of repolarizing TAMs to M1 phenotype is CP-870,893, which is an agonist monoclonal antibody (mAb) of CD40 [150, 151]. CD40 belongs to the tumor necrosis factor (TNF) family and it is broadly expressed in immune cells, including macrophages. CD40-activated macrophages are indicative of M1 phenotype correlating with reinforced proinflammatory cytokines release as well as upregulated expression of antigen presentation molecules such as major histocompatibility complex (MHC)-II [152]. According to Robert H.’s study, the administration of CD40 mAb in mice was able to induce macrophage-dependent tumor regression [146]. The tolerance and activity of CP-870,893 either as a single agent or in combination with chemotherapy have been tested in several clinical trials. In the first-in-human study, a single infusion of CP-870,893 was well tolerated at the 0.2 mg/kg. Partial responses (PR) were achieved in four patients with metastatic melanoma, and one of those four patients remained in partial remission even at the 14th month [126]. What is more, in patients with advanced PDAC, CP-870,893 administration with gemcitabine was revealed to induce an objective response rate (ORR) of 19% (4 in 23 patients developed a partial response), a median progression-free survival (mPFS) of 5.6 months, and a median overall survival (OS) of 7.4 months, which are superior to the historical efficacy of single gemcitabine in PDAC (ORR of 5.4%, mPFS of 2.3 months, and mOS of 5.7 months) [127, 146]. Anyway, those clinical trials are still at an early stage with small sample size [126, 127, 146, 153]. Further randomized clinical studies with larger sample size are definitely warranted to validate their potential in clinical applications.

### Agents inhibiting macrophages recruitment

As mentioned above, most of the TAMs originate from the bone marrow monocyte procurers. Recruitment of TAMs to the tumor sites or PMNs is a consequence of the continuous presence of tumor-derived chemoattractants. Therefore, cutting off those attracting signals for the macrophage recruitment appeals to be another promising solution for TAMs targeting anti-cancer therapeutic approach.

In addition to their roles in educating macrophages into M2 phenotype, both CSF-1 and CCL2 are responsible for recruiting TAMs into TME. It was reported that both small molecular inhibitors and antibodies targeting either CCL2/CCR2 or CSF-1/CSF-1R signaling axis obviously inhibited the mobilization of monocytes and macrophages accumulation in tumor sites. As a matter of fact, several inhibitors and antibodies targeting the TAM recruiting factors are being evaluated in early clinical trials across various types of tumor [132, 133, 154, 155]. For example, emactuzumab (RG7155) is a novel humanized antibody targeting CSF-1R in both ligand-dependent and ligand-independent manners [154]. Researchers found that administration of RG7155 significantly lowered the amount of CSF-1R expressing TAMs in on-treatment biopsies from tumor lesions [154]. A similar promising result has also been reported from clinical achievements in diffuse-type giant cell tumor (Dt-GCT), a neoplastic disorder characterized by CSF-1 overexpression and CSF-1R-positive TAM accumulation. In this study, among the 28 patients totally enrolled, 24 cases (86%) achieved complete response (CR) or PR, and three patients (11%) had stable disease (SD), with the average duration of response over 1.9 years [129]. However, whether this inspiring result in Dt-GCT could be carried over to other solid tumors remains a question and requires further investigation. What is more, pexidartinib (also known as PLX3397), an oral tyrosine kinase inhibitor of CSF-1R, exhibited similar efficiency (PR 52%, SD 30%, progressive disease 4%) in Dt-GCT patients as what RG7155 exhibits [130]. However, the phase II clinical trial showed no benefit from the administration of pexidartinib in 38 recurrent GBM patients [130]. But it is still worth looking forward to the results of many other ongoing clinical trials, which are conducted in c-kit-mutated melanoma, prostate cancer, sarcoma, and etc. [130]. Encouragingly, preliminary clinical benefit has been observed in a phase Ib trial evaluating the safety and effectiveness of CCX872, an orally administered CCR2 inhibitor, in patients with advanced pancreatic cancer. According to the data announced in January 2018, 29% patients receiving CCX872 and FOLFIRINOX combination therapy survived at the 18th month, more favorable than previously published OS rates of 18.6% at 18th month using FOLFIRINOX alone [132, 133]. Furthermore, a number of agents, such as CCL2 inhibitor bindarit, anti-CCL2 mAb carlumab, CSF1 inhibitor GW2580, and dequalinium-14, have been confirmed of potent and sustained anti-tumor activities via declining macrophages infiltration in a battery of cell lines and xenograft models [156–160]. It is conceivable that some of these agents will enter clinical trials in the near future to be further evaluated for their safety profiles and benefits in patient cohorts [155].

## Conclusions and perspectives

Cancer is more of a systemic disease since metastasis occurs in the majority of patients. Effectiveness achieved by existing therapeutics is far from satisfactory, since most of the current paradigms are designed to eliminate or interdict tumor cells themselves while the successful outgrowth of metastases is largely influenced by non-malignant cells of the tumor microenvironment (TME) [5, 6, 82]. As the major orchesters of the TME, TAMs tightly regulate tumor metastasis in all of the steps involved. In this review, we discussed the implicated regulation factors participating in recruitment and polarization of TAMs. In specific, we detailedly described the underlying mechanisms for TAM-involved tumor metastasis. When we get a better understanding of the correlation between TAMs and metastasis, the potential therapeutic strategies targeting TAMs would display a promising picture for cancer intervention. Indeed, we believe that targeting the pro-metastatic components of TME and rebuilding a healthier microenvironment with a reborn capacity to hamper tumor growth will definitely hold promise for cancer therapy.

In the past decades, our mechanistic investigations of TAMs never ceased and several TAM-targeted agents are available nowadays. Although TAM-targeted therapy based on modulation of TAM survival, polarization, and recruitment is attracting more and more attention in cancer prevention and treatment, there are many fundamental hurdles lying ahead before the findings of those researches finally transmitted into clinical benefits.

Firstly, TAMs are endowed with remarkably heterogenous roles in modulating metastasis. On the one hand, while TAMs are conventionally acknowledged as M2-like, they can, in fact, exhibit phenotypes anywhere in between tumoricidal M1 type and pro-tumoral M2 type. How phenotypes switch over the course of tumor progression is not fully known. On the other hand, molecular and cell-biological details involved in promoting metastasis might be more complicated than what we expect. Various major points of regulation networks remain elusive. Therefore, it is of great necessity for us to explore the unknown mechanisms underlying TAM-facilitated metastasis and figure out more detailed TAM characterizations as well as associated molecular profiles in TME.

Secondly, in spite of inspiring preclinical data obtained from numerous laboratories, the translational benefits of agents targeting TAMs are somewhat not that satisfactory in clinical studies. No agent has received official approval for clinical use of cancer treatment so far [161, 162]. There is an intriguing possibility that tumors with different histological types and gradings, different genetic background, as well as diverse local inflammatory profiles, might have heterogenous responses to the same treatment. Therefore, there arises the tip of a far larger iceberg: what histology types or what cellular and molecular features in TME would benefit from TAM-targeted therapy? The answer is pending. Further explorations in both preclinical and clinical studies are in desperate need. In clinical practice, pathology reports do not routinely describe TAM features in tumor samples, making it difficult to identify potential TAM-target beneficiaries and creating a gap in knowledge between the clinic and tumor immunology research. Hence, figuring out TAM-related features, such as amount, phenotypes, and cytokine profiles on the pathology reports, or even assessing circulating M2 macrophage numbers as well as systemic CSF1, CCL2 levels might provide a tool for better predicting cancer metastasis and stratifying patients [158]. Furthermore, TAM-targeting therapies, either by blocking their infiltration into TME or by impairing pro-tumoral functions, are insufficient to achieve satisfying metastasis control without a direct attack on tumor cells. Approaches combining TAM-targeting agents with chemotherapeutics, irradiation, antiangiogenic agents, and immune checkpoint inhibitors may pave the way for augmented control of progression and metastasis [163, 164]. But most of these concerns have not been realized in a clinically significant way. Further studies are warranted to evaluate their therapeutic effectiveness both as a single agent or as part of a combination therapy.

When we come to talk about the immune checkpoint based therapy, it is worth noting that targeting immune checkpoint pathways, such as the innate anti-phagocytic axis of CD47-SIRPα (signal-regulatory protein alpha) pathway and LILRB receptor pathway, is emerged as one of most attractive strategy for cancer therapy. For example, CD47 expressed in tumor cells can interact with signal-regulatory protein alpha (SIRPα) which is a transmembrane protein on macrophage and the main receptor of CD47, thereby delivering the “do not eat me” signals to macrophages [165]. Studies found that the expression of CD47 increases in various tumors to evade immune attack [166]. Therefore, CD47-SIRPα interaction blockade by anti-CD47 blocking antibody increased the infiltration of macrophages in the TME, thus promoting phagocytosis of CD47^**+**^ tumor cells to exert antitumor efficacy [167, 168]. Besides, the leukocyte immunoglobulin-like receptor B (LILRB) family members are negative regulators of myeloid cell activation [169, 170]. Studies found that LILRB2 blockade by LILRB2-specific monoclonal antibodies effectively polarized macrophage cells toward an inflammatory phenotype and enhanced pro-inflammatory responses, thus acting as a myeloid immune checkpoint by reprogramming TAMs and provoking antitumor immunity [171, 172].

Thirdly, noting that TAMs do not exert functions in isolation, the TME is a complex system consists of a plethora of cells other than TAMs, such as fibroblasts, epitheliums, neutrophils, mesenchymal stem cells, myeloid cell-derived suppressor cells, and mast cells. They and their stroma around are tightly linked and interacted with each other constantly alongside the formation of metastasis [117]. Preclinical experiments targeting TAMs without the consideration of intricacy and versatility in their interactions are prone to fail in arising effective therapeutic approaches in the clinic. Hence, digging into the respective roles of those components of TME and modeling their intricate interactions evolving along with the metastasis by system biology approaches may be the avenues for future research [162].

In conclusion, this review provides an overview of our current understanding of the cross talk between TAMs and tumor cells during tumor progression, particularly in metastasis. As stated above, TAM represents a novel and attractive target that may alter the landscape of future cancer therapy, although many critical obstacles are still lying ahead and more endeavors in this aspect are needed to be done.

## Data Availability

Not applicable.
